# Assessing the generalizability of artificial intelligence in radiology: a systematic review of performance across different clinical settings

**DOI:** 10.1097/MS9.0000000000004166

**Published:** 2025-10-22

**Authors:** Muhammad Umer Suleman, Muhammad Mursaleen, Umer Khalil, Abdul Saboor, Muhammad Bilal, Shahbaz Azam Khan, Muhammad Ali Subhani, Muhammad Ali Hussnain, Syeda Nafisa Tabassum, Maryum Tahir

**Affiliations:** a Department of Internal Medicine, Ayub Medical College, Abbottabad, Pakistan; b Bangladesh Rural Advancement Committee: BRAC, Dhaka, Bangladesh

**Keywords:** artificial intelligence, convolutional neural networks (CNNs), deep learning, diagnostic accuracy, diagnostic radiology, external validation, generalizability, generative adversarial networks (gANs), reproducibility of results

## Abstract

**Introduction:**

Artificial intelligence (AI) applications in diagnostic radiology have demonstrated remarkable accuracy on institutional datasets. However, concerns about external generalizability performance when models encounter data from different hospitals, scanners, or patient populations remain a major barrier to clinical deployment.

**Methods:**

We performed a Preferred Reporting Items for Systematic Reviews and Meta-Analyses and Assessing the Methodological Quality of Systematic Reviews -compliant systematic review of peer-reviewed studies (January 2022 – June 2025) reporting both internal and external validation of AI diagnostic models applied to computed tomography, magnetic resonance imaging, or X-rays. The review was registered in the PROSPERO database. PubMed and Embase searches identified 342 records; after de-duplication, screening, and eligibility assessment, six studies met our inclusion criteria. These studies addressed diverse diagnostic tasks using deep learning architectures (3D Convolutional Neural Networks, Generative Adversarial Networks-augmented models, no-new-U-Net ensembles, and regulatory-cleared systems).

**Results:**

Internal-validation area under the curve (AUC) ranged from 0.76 to 0.95; sensitivities were generally >85% and specificities >68%. In external validation, performance declined modestly in AUC (median drop ~0.03), with larger decreases in specificity (up to ~24 percentage points). Quality Assessment of Diagnostic Accuracy Studies – Version 2 assessment revealed low overall risk of bias in five studies; one study had high patient-selection bias, and another had unclear sampling. Methods that appeared to enhance generalizability included multicenter training, data augmentation with generative adversarial networks, and incorporation of clinical variables.

**Conclusion:**

AI models in radiology tend to underperform on external data despite strong internal performance. Mandatory external validation on diverse cohorts and cautious clinical integration are recommended.

## Introduction

Artificial intelligence (AI), particularly deep learning, has rapidly transformed medical imaging. Convolutional Neural Network-based algorithms (CNN) now automate many radiology tasks such as lesion detection, classification, and quantification with performance often comparable to radiologists^[1]^. This promise has spurred extensive development and regulatory interest. For example, a recent analysis found 691 Food and Drug Administration (FDA)-cleared AI/Machine Learning (ML) medical devices (as of late 2023), with a clear dominance of radiology applications due to the abundance of routinely acquired imaging data^[2]^. These developments underscore AI’s potential to enhance diagnostic efficiency and accuracy in radiology.

**HIGHLIGHTS**

- Artificial intelligence models had strong internal area under the curve (AUC) but lost up to 24% specificity externally.
- Generative adversarial network-based augmentation and multicenter training improved generalizability.
- Generative adversarial network use increased one model’s external AUC from 0.836 to 0.933.
- Sensitivity stayed above 85% in most external validations.
- External validation and local testing are essential before clinical use.

Despite these advances, clinical translation faces a critical challenge: models may not generalize to new data. In practice, AI systems frequently encounter domain shifts, differences in patient demographics, disease prevalence, scanner hardware, and imaging protocols when applied at a different center^[3,4]^. Numerous studies have shown that performance often degrades under such shifts. For example, one systematic review of 86 deep-learning algorithms in radiology reported that 81% exhibited decreased accuracy on external datasets, with nearly a quarter experiencing a substantial drop [≥0.10 area under the curve (AUC)]^[5]^. Domain shifts are a key culprit: as Musa *et al* note, “Variations in patient demographics, imaging protocols, equipment, and environmental factors can lead to significant performance degradation” for models applied to a new population^[6]^. In other words, a model trained on one hospital’s data may not maintain accuracy at another. This gap between internal (development) performance and external validity undermines trust and raises safety concerns for clinical deployment. Equity and bias further complicate generalizability. Deep models can inadvertently learn demographic “shortcuts” from imaging data^[7]^. For instance, Yang *et al* showed that radiology AI can predict patient race from chest X-rays and that models relying on these demographic cues perform worse in new settings. They found that models with less dependence on demographic attributes tended to be more “globally optimal,” retaining better accuracy and fairness across different test populations^[7]^. Indeed, previous work has documented disparities (e.g., chest X-ray models underdiagnosing Black patients^[7]^) when AI is applied to diverse cohorts. These findings highlight that poor generalization often goes hand in hand with bias, and assessing model performance on out-of-distribution populations is essential for equitable care.

Improving robustness requires overcoming data heterogeneity. Medical imaging includes multiple modalities [X-ray, magnetic resonance imaging (MRI), computed tomography CT, ultrasound, and PET], each providing unique anatomical and pathological insights^[8]^. Recent studies suggest that incorporating diverse data sources can bolster generalization. For example, Ozkan and Boix demonstrated that a “multi-domain” model trained on a mix of modalities and image views achieved superior generalization (up to +8% accuracy) on out-of-distribution tasks compared to modality-specific models^[8]^. Similarly, federated learning, which involves collaborative training across multiple hospitals without sharing raw images, has shown promise. In a large chest X-ray study (610 000 images across five institutions), models trained jointly outperformed those trained at single sites on external test data^[9]^. In other words, exposure to more heterogeneous training data (via multi-center or data-augmentation techniques) appear to mitigate performance drops from domain shifts.

The recognition of these challenges has led to new reporting standards. Reporting guidelines like Transparent Reporting of a Multivariable Prediction Model – AI Extension (TRIPOD-AI) and Developmental and Exploratory Clinical Investigations of Devices – AI Extension (DECIDE-AI) explicitly call for transparent validation and external testing of AI systems^[10]^. However, most radiology AI studies published to date have focused on single-institution development, and few syntheses have quantified generalizability across different clinical settings. To address this gap, we performed a comprehensive systematic review [following Preferred Reporting Items for Systematic Reviews and Meta-Analyses (PRISMA) guidelines] of AI models in diagnostic radiology with external validation results. We aimed to quantify how much performance degrades in external settings, to evaluate study quality [using Quality Assessment of Diagnostic Accuracy Studies – Version 2 (QUADAS-2)], and to identify strategies that improve cross-site robustness. By focusing on the recent literature (2022–2025), we capture the latest advancements and remaining obstacles in AI generalizability in radiology.

## Methods

We conducted this systematic review following the PRISMA guidelines^[11]^. The work has been reported in line with the Assessing the Methodological Quality of Systematic Reviews (AMSTAR) guidelines^[12]^. This review was registered in the PROSPERO database.

### Search strategy

We searched PubMed and Embase (via Embase.com) for English-language articles published between 1 January 2022 and 10 June 2025, combining MeSH and free-text terms for AI, radiology modalities, diagnostic tasks, and validation type. These two databases were chosen because they comprehensively index the vast majority of peer-reviewed literature in radiology, AI, and clinical medicine. The search combined MeSH and free-text terms for AI (“artificial intelligence” OR “deep learning” OR “machine learning”), radiology (“radiology” OR “medical imaging” OR “CT” OR “MRI”), diagnostic tasks (“diagnosis” OR “detection” OR “classification”), and validation (“external validation” OR “multicenter”), intersected with “internal validation.” We also screened reference lists of identified studies for additional reports. Two reviewers independently screened titles and abstracts, and then full texts, resolving conflicts by consensus.

### Inclusion criteria

The inclusion criteria for this review encompassed peer-reviewed studies published between 2022 and 2025 that involved human subjects. Eligible studies specifically focused on the diagnostic application of AI using CT or MRI data. To ensure methodological rigor, studies were required to report both internal validation, such as cross-validation or hold-out testing within the training institution, and external validation using an independent dataset from a different site or population. External validation refers to testing the performance of AI models on independent datasets that were not used during model development. This is crucial for assessing the generalizability of AI systems across various clinical settings^[13,14]^. For this review, external validation involved evaluating AI models on datasets from different institutions, patient populations, and imaging protocols to ensure that the models’ performance was not overly tailored to specific conditions. Dataset diversity, including multi-center inclusion and variation in patient demographics and scanner models, is critical to test whether the model can perform reliably in diverse real-world scenarios^[13,14]^. Studies with external validation were prioritized to capture these variations and evaluate how well the AI models generalized across different clinical environments. We focused on studies that reported the AI models used and their corresponding diagnostic tasks. We prioritized studies that provided sufficient details about the model architecture and the specific diagnostic applications to ensure consistency and comparability of the AI systems reviewed. Additionally, studies had to present quantitative performance metrics, including at least one of the following: AUC, sensitivity, specificity, or accuracy.

### Exclusion criteria

The exclusion criteria eliminated studies that involved phantom models or animal subjects, as the review focused exclusively on human-based research. We limited our review to studies using CT and MRI because these modalities were the most commonly employed in diagnostic radiology and have been extensively researched in AI applications. Research involving X-rays, ultrasounds, and PET scans was excluded due to the lack of sufficient data or published studies that did not meet our inclusion criteria, particularly with respect to both internal and external validation metrics. Studies were also excluded if they addressed tasks such as image segmentation, workflow optimization, or other non-diagnostic applications that did not include classification metrics. Additionally, studies lacking external validation, defined as testing on an independent dataset from a different site or population, were excluded.

### Data extraction

From each included study, the following data were extracted: the study citation, year of publication, and country of origin; the AI model architecture used along with its training strategy; the imaging modality employed (either CT or MRI) and the specific target diagnosis addressed. Additionally, details regarding dataset sizes and characteristics were collected for both internal and external validation cohorts. Performance metrics were recorded, including AUC with 95% confidence intervals where available, as well as sensitivity, specificity, accuracy, and inference time. Lastly, any strategies employed to enhance generalization, such as data augmentation or the use of multicenter datasets, were also noted.

### Risk-of-bias assessment

We assessed the methodological quality of each included study using the QUADAS-2 tool, which evaluates four key domains^[15]^. The first domain, Patient Selection, considered whether studies used consecutive or selective sampling methods. The second domain, Index Test, examined whether the AI model’s threshold was pre-specified and whether blinding was applied during interpretation. The third domain, Reference Standard, assessed the validity of the diagnostic standard used, such as histopathology, reverse transcription polymerase chain reaction, or expert consensus. The final domain, Flow and Timing, evaluated whether all eligible patients were included and whether the time interval between imaging and reference standard assessment was appropriate. Each domain was rated as having a “Low,” “Some concern,” “High,” or “Unclear” risk of bias, with assessments independently conducted by two reviewers.

## Results

**Figure 1.**
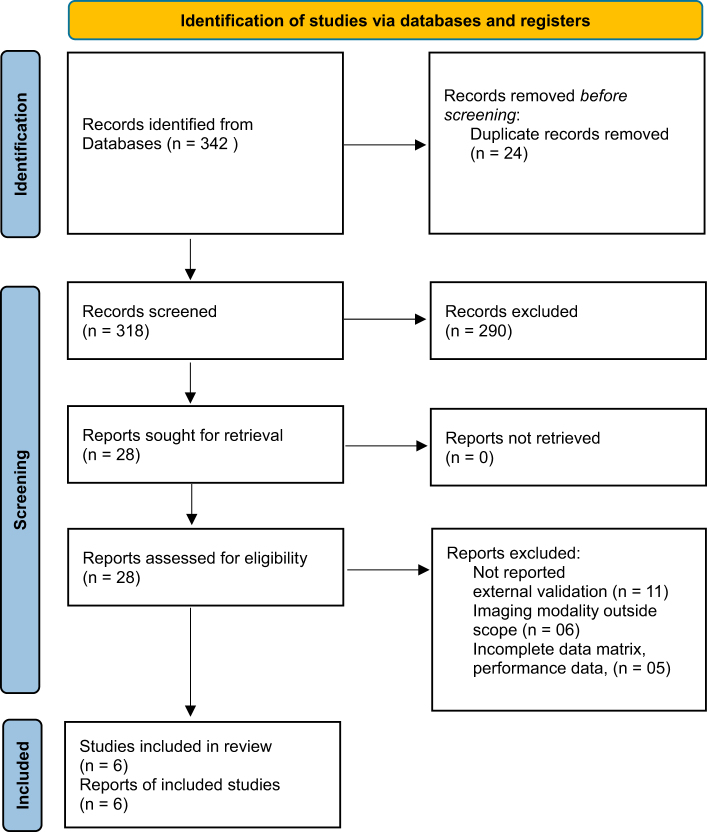
PRISMA 2020 flow diagram illustrating the study selection process.

Our search yielded 342 records. After removing 24 duplicates, 318 titles/abstracts were screened. Of these, 290 were excluded (211 did not use external validation, 50 were outside the radiology domain, and 29 lacked sufficient metrics), leaving 28 full-text articles for review. Twenty-two full-text articles were excluded (11 lacked external validation, 6 addressed non-CT/MRI modalities, and 5 omitted key metrics), resulting in six studies that met all inclusion criteria (Fig. 1).

### Study characteristics

The six included studies (published 2022–2025) spanned a variety of clinical tasks and AI architectures (Table 1). Imaging modalities included chest CT (COVID-19 vs influenza vs normal classification)^[16]^, prostate MRI (cancer detection)^[17]^, whole-body CT (bone metastasis classification)^[18]^, brain MRI (multiple sclerosis lesion detection)^[19]^, nasopharyngeal MRI^[20]^, and cervical spine CT (trauma fracture detection)^[21]^. Models ranged from a 3D Inception CNN and a commercial computer-aided detection/diagnosis system to generative adversarial network (GAN)-augmented CNNs and nnU-Net ensembles^[16–21]^.

**Table 1:** Study characteristics

Study (year)	Modality	Diagnostic task	Model	Internal metrics	External metrics
Vaidyanathan *et al*, 2022	Chest CT	COVID-19 vs influenza vs normal	3D inception CNN	AUC 0.91 (CI 0.89–0.93); Sens 87.9%; Spec 87.2%	AUC 0.90 (CI 0.87–0.92); Sens 83.4%; Spec 91.2%
Giganti *et al*, 2025	Prostate MRI	Clinically significant prostate cancer (Gleason ≥2)	CE-marked DL CAD	AUC 0.95; Sens 96%; Spec 68%	AUC 0.91; Sens 95%; Spec 67%
Zhang *et al*, 2025	Whole-body CT	Bone metastases classification	3D lesion-classification CNN	Accuracy 92.3%; BalAcc 86.8%	Accuracy 91.1%; BalAcc 83.4%
Brugnara *et al*, 2024	Brain MRI	Multiple sclerosis lesion detection	CNN + GAN-augmented training	AUC 0.95 (with GAN)	AUC 0.933 (with GAN); 0.836 (no GAN)
OuYang *et al*, 2023	Nasopharynx MRI	Clinically significant prostate cancer detection	nnU-Net + classifier	AUC 0.92; Sens 79.5%; Spec 91%	AUC 0.88; Sens 74.3%; Spec 92.8%
Harper *et al*, 2025	Cervical CT	Trauma-related fracture detection	RSNA-challenge CNN	Sens 85%; Spec 94%	Sens 86%; Spec 70%

AUC, area under the curve (ROC curve); BalAcc, balanced accuracy; CNN, convolutional neural network; DL CAD, deep learning computer-aided diagnosis; GAN, generative adversarial network; nnU-Net, No-new-Net (a self-configuring deep learning segmentation framework); RSNA, Radiological Society of North America; Sens, Sensitivity; Spec, Specificity.

**Table 2:** Factors influencing generalizability of AI in radiology as identified in included studies

Generalizability factor	Impact on external performance
Population shift (e.g., trauma and age)	↓ Specificity^[21]^
Modality change or protocol variation	↓ Specificity or AUC^[16,18,21]^
Multicenter training	↑ Stability^[17,18]^
GAN-based augmentation	↑ External AUC^[17]^

AI, artificial intelligence; AUC, area under the curve; GAN: generative adversarial network.

Internal-validation performance was uniformly strong: AUCs ranged from 0.76 to 0.95 across tasks; reported sensitivities were generally ≥85% and specificities ≥68%. In external validation, performance was consistently lower (Table 1). The median AUC drop was approximately 0.03; for example, Giganti *et al* observed an AUC fall from 0.95 internally to 0.91 externally^[17]^. Notably, specificity often declined more than sensitivity. Harper *et al* reported nearly unchanged sensitivity (85%→86%), but specificity fell from 94% to 70% on an older trauma cohort^[21]^. Brugnara *et al* found that GAN-based augmentation preserved external AUC (0.933 with GAN vs 0.836 without)^[19]^. Overall, external validation showed modest median decreases in accuracy and AUC (Table 1).

We observed that larger, multi-center training cohorts tended to yield smaller performance gaps. For example, Giganti *et al* and Zhang *et al* (both using multicenter MRI datasets) reported only minor accuracy reductions during external validation^[17,18]^. In contrast, the smallest study (Harper *et al*) had the largest specificity drop^[21]^. All studies used retrospective validation; however, five out of six tested models were tested on truly independent external cohorts from different institutions or patient groups.

All studies used retrospective validation; most validated on external centers or different populations. External performance dropped in most cases, particularly specificity^[21]^. GAN-based augmentation improved external generalizability^[19]^. Multicenter cohorts showed smaller drops between internal and external metrics^[17,18]^.

### Dataset and protocol differences

Internal datasets ranged from 144 to 1271 cases, while external cohorts ranged from 120 to 1247 cases^[16–21]^. Some original study authors noted differences in scanner vendor or patient demographics between their internal and external datasets, which they suggested may have influenced performance^[16–21]^. These observations were not uniformly quantified across studies but highlight factors potentially affecting generalizability. Two studies additionally reported inference times: Vaidyanathan *et al’s* model required ~56 s per scan^[16]^, whereas Harper *et al’s* CNN took ~ 6.4 s per exam^[21]^. No study reported prospective clinical deployment; all were retrospective evaluations.

### Risk-of-bias assessment (QUADAS-2)

**Figure 2.**
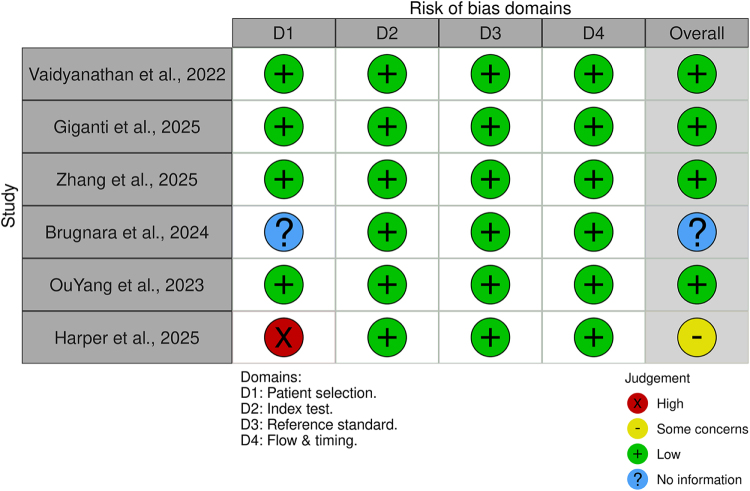
Risk of bias assessment (QUADAS-2).

QUADAS-2 assessment (Fig. 2) indicated generally low risk of bias. Five studies had overall low risk, with low risk in all domains (consecutive patient selection, pre-specified models, valid reference standards, and complete flow)^[16–20]^. Harper *et al* was an exception: its case-control design (50 fracture vs 50 non-fracture cases) led to high risk in Patient Selection^[21]^. Brugnara *et al* had unclear risk in Patient Selection due to limited sampling details^[19]^. No study showed high bias in the Index Test or Reference domains. We note that most algorithms were applied uniformly across patients without outcome-driven recalibration, mitigating index-test bias.

Most studies used retrospective cohorts; selection bias was low in prospective or consecutive sampling^[17,20]^ but higher in case-control designs^[21]^. Index tests had predefined models without data-driven thresholds, and reference standards were generally gold-standard (histology or consensus), so bias in those domains was low. All studies applied the AI to all eligible cases without exclusion, minimizing flow bias.

### Consistent internal–external performance drop

Across all six studies, external validation performance was consistently lower than internal validation, most notably in specificity. Harper *et al* reported stable sensitivity (85% vs 86%) but a notable drop in specificity from 94% to 70% when their CNN was tested on a new trauma cohort of older patients^[21]^. Giganti *et al* demonstrated strong generalizability of a prostate MRI classifier, but external AUC still declined slightly from 0.95 to 0.91, despite a robust multicenter design^[17]^. Zhang *et al* reported a minor drop in classification accuracy from 92.3% to 91.1% across different hospitals, suggesting that high-quality multicenter training can buffer generalizability losses^[18]^. This drop, though sometimes modest, reflects a consistent pattern: AI models often fail to replicate internal performance when applied to new populations, scanners, or institutions.

### Setting and population impact generalizability

Several study authors suggested that external performance differences may have been related to patient population or clinical environment. For example, Harper *et al* trained on a standard dataset but validated externally on an elderly trauma population^[21]^. Their model’s specificity suffered, possibly due to anatomical changes or scan quality differences in older adults. These cases highlight that even when external data are available, differences in disease prevalence, imaging protocols, or demographic factors can reduce model reliability. While these observations are qualitative rather than systematically quantified in this review, they illustrate how demographic or protocol variations can affect model reliability.

### Techniques that improved generalizability

Several studies demonstrated methods that helped mitigate performance drops during external validation. Brugnara *et al* showed that training with GAN-generated synthetic lesions significantly improved external AUC from 0.836 (without augmentation) to 0.933 (with GAN-augmented data)^[19]^. This suggests that data augmentation can help simulate inter-institutional variation. Giganti *et al* and Zhang *et al* used multicenter training and testing with harmonized protocols, leading to smaller internal-external gaps^[17,18]^. For example, Zhang *et al’s* classification accuracy dropped only ~1.2% externally despite using five hospital datasets^[18]^. Thus, diverse training data, whether synthetic or multicenter, appears crucial for achieving robust generalization. These are qualitative insights drawn from individual study reports rather than standardized metrics extracted in this review.

### Imaging modality considerations

The reviewed studies spanned CT (three studies) and MRI (three studies). No major differences in generalizability were directly attributable to modality; rather, the key factor appeared to be model exposure to heterogeneous data during training. CT-based models tended to show sharper specificity drops in external settings, likely reflecting protocol and hardware differences^[16,18,21]^. MRI models, particularly those trained on standardized data across centers, showed more stable external performance, though that may reflect stricter design and clinical task (biopsy-based labels)^[17,19,20]^.

### Real-world applicability remains cautious

Despite generally strong external AUCs (most ≥0.90), the clinical use of AI models still requires caution. None of the studies conducted a prospective deployment in live clinical settings. Few studies reported inference time or integration into radiologist workflows, though Harper *et al* (6.4 s/exam) and Vaidyanathan *et al* (~56 s/scan) did provide estimates^[16,21]^. Even with regulatory clearance (as in Giganti *et al’s* Conformité Européenne (CE)-marked tool), threshold tuning, patient mix, and scanner variation must be managed locally to maintain performance^[17]^.

### Overall synthesis

Generalization of AI in radiology is possible, but fragile. Strong internal metrics often do not transfer unless external data diversity is accounted for. Performance drop is usually modest in sensitivity, but specificity and calibration frequently suffer. As summarized in table 2, strategies like multicenter training, augmentation, and model transparency (e.g., interpretability) should be standard in future work.

## Discussion

This systematic review evaluated recent radiology AI studies that reported both internal and external validation. Despite strong performance in development cohorts, all six models underperformed to some extent on independent data, mirroring trends reported in larger analyses^[5]^. The consistent finding across studies was that external AUC and accuracy were slightly lower, with the largest drops often in specificity^[16–21]^. This reinforces that internal performance does not guarantee real-world performance; an AI model may overfit to site-specific features.

Our findings corroborate prior evidence of limited generalizability in medical imaging AI. Yu *et al* found that the majority of deep-learning models lose accuracy on external validation^[3]^, and our six-study sample likewise showed ubiquitous performance declines. Several factors were implicated in the loss of generalizability. Domain shift, i.e., differences in data distributions between training (development) and target settings, was a dominant theme as explained in Musa *et al*^[6]^. Domain shift may arise from changes in patient populations, disease prevalence, or image features^[6]^. In practice, such differences may manifest as shifts in the prevalence of disease or image appearance that the model was not exposed to during training. For example, Harper *et al* applied a trauma fracture classifier trained on younger patients to an older cohort and observed a large specificity drop^[21]^. Population heterogeneity is another source of shift: if training data lack demographic diversity, models may overfit to the represented subgroups and underperform on underrepresented groups^[7,22]^. For instance, AI models have been documented to under-diagnose certain racial groups in imaging studies^[7]^. All included studies noted demographic or disease-prevalence differences between sites, and such heterogeneity likely contributed to reduce external AUC.

On the technical side, overfitting and under-specification were identified as key obstacles to generalization. Overfitting, where a model learns idiosyncrasies of its training set, leads to inflated internal accuracy that does not transfer. Under-specification (multiple models fitting the same training data but diverging on new data) similarly undermines reliability. In practice, deep neural networks often produce overconfident predictions on new data if not explicitly calibrated or regularized. In our review, all six studies demonstrated stronger performance on internal validation than on external testing. For instance, Zhang *et al* reported a decrease in balanced accuracy when their CT model for bone metastasis was tested on external data from different hospitals^[18]^. Giganti *et al* observed a small but consistent drop in AUC when applying a prostate MRI classifier across centers with varying scanners^[17]^. Brugnara *et al* noted improved external AUC following GAN-based augmentation, suggesting that model performance was sensitive to image domain differences^[19]^. These findings confirm that domain shift, driven by variability in scanner hardware, imaging protocols, and institutional sources, is a key driver of reduced generalizability in radiology AI, alongside training overfitting and poor calibration.

What strategies helped? Multi-site training emerged as beneficial. Giganti *et al* and Zhang *et al* both used multicenter data and reported only marginal performance drops^[17,18]^. Federated or pooled training effectively increases the diversity of the training set, thus reducing overfitting to one site^[9]^. GAN-based augmentation also showed promise: Brugnara *et al*’s GAN-augmented CNN had a higher external AUC than the standard model, consistent with ideas that synthetic data can fill gaps in representation^[19]^. These results echo recent research: Ozkan and Boix demonstrated that multi-domain models generalize better on limited-data tasks^[8]^, and Arasteh *et al* showed that federated learning substantially boosts out-of-distribution accuracy^[9]^.

This work highlights the urgent need for rigorous validation practices. Echoing guidelines like TRIPOD-AI and DECIDE-AI, we emphasize that new AI models should be tested on external, preferably prospective cohorts before adoption^[10]^. The TRIPOD-AI statement (for predictive model development) explicitly calls for transparent reporting of data sources, modeling methods, and rigorous validation, including external datasets. Similarly, DECIDE-AI – a new consensus guideline for early-stage clinical evaluation warns that “small changes in the underlying data distribution” (dataset shift) can cause large performance variability and “unexpected, potentially harmful outcomes”^[23]^. DECIDE-AI recommends planning studies to test generalizability across sites and patient cohorts^[23]^. Our reviewed studies generally fell short of these ideals: most were retrospective and did not prospectively evaluate integration. In future work, adherence to TRIPOD-AI and DECIDE-AI principles would strengthen evidence of real-world utility, for example, by including a planned multisite external validation and calibration analyses.

From a regulatory perspective, both the FDA and WHO emphasize the need for external validation and monitoring. The FDA’s recent AI/ML Software as a Medical Device action plan (and accompanying Good Machine Learning Practice guidelines) highlights iterative validation and risk-based evaluation through the product lifecycle^[24]^. For instance, the FDA now publishes draft guidance recommending explicit “predetermined change control plans” and transparency measures for AI devices^[24]^. These reflect recognition that static model approval is insufficient without accounting for domain shifts after deployment. The WHO, similarly, calls for appropriate validation with external data to ensure quality and safety of AI systems^[25]^ and for rigorous pre-release evaluation to minimize bias^[25]^. In practice, our review suggests that regulators should require evidence that radiology AI tools perform well across the intended range of scanners and patient populations, not just in a lab setting.

Real-world deployment raises additional concerns. Inference time and hardware requirements can hinder practical use: deep CNNs often need Graphics Processing Unit (GPU) acceleration and may not run in real-time on standard hospital systems. One review notes that implementing AI requires *high-performance hardware* and robust software integration into Picture Archiving and Communication System (PACS)/Radiology Information System (RIS) to avoid workflow disruptions^[22]^. Another challenge is the calibration of model outputs. As Mehrtash *et al* demonstrated, modern deep networks can be poorly calibrated and overconfident on unseen data^[26]^, which is dangerous in practice. Techniques like ensembling or Monte Carlo dropout can improve calibration and help the system flag unfamiliar cases^[26]^. Workflow integration also entails human factors; radiologists must be trained to interpret AI results, and the system should enable easy query or a second opinion. Any increase in reading time or alert fatigue can negate the benefit. A recent Mayo Clinic commentary emphasizes that AI adoption requires considering “operational, technical, clinical, and regulatory” hurdles as a whole^[27]^. Finally, equity is paramount: models may systematically underperform on minority groups, as Yang *et al* have shown^[7]^. Ensuring AI does not worsen disparities means validating and possibly re-training models on diverse demographic cohorts, as well as being transparent about any performance gaps.

Developers should aim for diverse training data (multiple centers and different populations) and robust design (pre-specifying thresholds and avoiding selective sampling) to minimize bias. Furthermore, performance should be reported transparently: alongside internal cross-validation, external test results should be provided, ideally with confidence intervals. Clinicians interpreting AI tools must be aware of these generalization gaps; an algorithm validated only internally may not achieve the same accuracy in a new hospital.

### Limitation

Several limitations temper our conclusions. The number of eligible studies was small (*n* = 6), reflecting that dual-validation radiology AI studies are still uncommon. The tasks and metrics were heterogeneous, precluding meta-analysis. This review primarily reflects CT and MRI literature, as eligible studies involving X-ray, ultrasound, or PET that met the strict inclusion criteria for internal and external validation were not available in the published evidence. Moreover, all included studies were retrospective; none reported prospective clinical trials. Thus, even low bias ratings (via QUADAS-2) do not guarantee real-world utility. We also only searched PubMed and Embase and English-language publications, so we may have missed some reports (although this field is dominated by English journals). Finally, our focus on 2022–2025 ensured currency but excluded older AI studies; however, earlier reports typically lacked external validation and would not change the finding of generally poor generalizability.

## Conclusion

This systematic review evaluated six peer-reviewed studies published between 2022 and 2025, each assessing the diagnostic performance of AI models in radiology through both internal and external validation. The findings demonstrate that while AI models often achieve high performance in internal datasets (e.g., AUCs ≥ 0.90), their performance frequently declines when applied to external data, particularly in specificity^[16–21]^.

The decline is often modest in sensitivity but more pronounced in specificity, indicating challenges in model calibration and generalizability when used in new clinical settings. Studies that incorporated multicenter validation, or data augmentation techniques (e.g., GANs), showed more stable performance across internal and external settings^[16–21]^. These results highlight both the potential and the limitations of current AI models in radiology.

While AI demonstrates clear advantages in speed and sometimes diagnostic accuracy, this review reaffirms that real-world clinical deployment requires rigorous external validation and sensitivity to patient, scanner, and protocol variability. No study in this review involved prospective implementation, reinforcing that generalization remains a critical barrier to clinical readiness.

## Recommendations

Based on the review findings, the following recommendations are proposed for researchers, developers, and regulatory bodies working on AI in radiology:

### For researchers and developers

- Prioritize external validation in every AI model development pipeline; single-site testing is insufficient for clinical claims.
- Use multicenter training datasets that reflect scanner, demographic, and disease variability to improve robustness.
- Implement data augmentation strategies, such as GANs, to simulate real-world diversity if external data access is limited.
- Ensure models are evaluated across different age groups, institutions, and equipment to uncover weaknesses in generalizability.
- Include metrics like calibration curves, not just AUC, to assess whether models maintain decision thresholds across datasets.

### For clinical integration

- AI tools should be integrated as decision support, not standalone diagnostics, until consistent generalizability is demonstrated.
- Local validation of pre-trained models should be mandatory before deployment, regardless of regulatory clearance.
- Inference time and workflow impact should be reported more consistently to assess usability in real-time clinical settings.

### For journals and regulators

- Require that studies include external validation before acceptance or approval.
- Encourage the use of standardized reporting frameworks (e.g., TRIPOD-AI and DECIDE-AI) to ensure transparency and replicability.

AI in radiology is advancing rapidly, and the results of this review are promising. However, the path to generalizable, clinically reliable AI remains challenging. By incorporating robust validation practices, model developers and clinicians can bridge the gap between AI research and its safe, effective use in real-world patient care.

## Data Availability

All data generated during this study are included in this published article.

## Research registration unique identifying number (UIN)

This study is registered in PROSPERO (CRD420251060155).
